# Molecular insights into shared and distinct etiologies of Alzheimer's disease and major depressive disorder at single‐nucleus resolution

**DOI:** 10.1002/alz70855_099627

**Published:** 2025-12-23

**Authors:** Michael W Lutz, Zhaohui Man, Ornit Chiba‐Falek

**Affiliations:** ^1^ Duke Department of Neurology, Durham, NC, USA; ^2^ Duke University School of Medicine, Durham, NC, USA

## Abstract

**Background:**

Late‐onset Alzheimer's disease (LOAD) patients often have comorbid neuropsychiatric symptoms (NPS) with depression and anxiety being most prevalent. Neuropsychiatric disorders including major depressive disorder (MDD) confer risk for LOAD later in life. Previously we identified shared genetic risk loci between LOAD and MDD. Here, we aimed to investigate in‐depth and compare the brain transcriptomic landscapes of LOAD and MDD at a cell‐type specific precision. Since MDD often develops early in life in contrast to LOAD, shared genetic etiology may reflect some of the earliest pathophysiological changes common to both diseases.

**Method:**

Single nucleus (sn)RNA‐seq data from two studies: ROSMAP for LOAD and the Douglas Research Center for MDD was analyzed, specifically, dorsolateral pre‐frontal cortex datasets for 157 LOAD cases and 143 controls vs 37 MDD cases and 34 controls. Differential gene expression (DEG) was performed using Nebula with case/control status based on clinical diagnosis of LOAD or MDD. Shared and district LOAD and MDD DEGs were catalogued. Intercellular communication networks were analyzed using CellChat.

**Result:**

FDR‐significant DEGs were found for multiple cell types. Common DEGs were found for GABAergic neurons (*n* = 101), Glutamatergic (*n* = 163) neurons and Interneurons (*n* = 15). Two DEGs were found for Microglia (*PARD3B, S100Z*). The genes in these cell types were downregulated in both LOAD and MDD cases. Pathway analysis for the DEGs, across these cell subtypes showed significant enrichment for terms related to oxidative phosphorylation, mitochondrial electron transport and synaptic signaling, suggesting mitochondrial dysfunction as a common biological process. In MDD and LOAD there was loss in cell‐cell communication (CCC) networks between astrocytes and other cell subtypes while in LOAD there was an overall gain in CCC between several of the neuronal subtypes.

**Conclusion:**

Our single‐cell analysis provides mechanistic insights into the shared and divergent molecular etiologies, dysregulated pathways, and impaired cellular communications between LOAD and MDD. This knowledge has a translational impact toward the identification of actionable targets as novel therapies to treat depression symptoms earlier in disease stages as a mean to delay or alleviate depression onset in LOAD.

